# Domestication Cultivation and Nutritional Analysis of *Hericium coralloides*

**DOI:** 10.3390/jof11110785

**Published:** 2025-10-31

**Authors:** Yun Li, Jiarong Cai, Xiaomin Li, Xin Hu, Junli Zhang, Xiaoping Wu, Junsheng Fu

**Affiliations:** 1 College of Life Sciences, Fujian Agriculture and Forestry University, Fuzhou 350002, China; 2 Tibet Academy of Agricultural and Animal Husbandry Sciences, Lhasa 850002, China

**Keywords:** *Hericium coralloides*, biological characteristics, nutritional composition, antioxidant properties, culture conditions, domestication and cultivation

## Abstract

*Hericium coralloides* is a valuable medicinal and edible mushroom renowned for its unique bioactive compounds. This study focuses on the isolation of a wild strain (SH001) exhibiting promising cultivation potential and health promoting properties. A wild fungal strain from the Tibetan Plateau was isolated and identified as a novel *H. coralloides* based on its morphological and molecular characteristics. The optimal growth conditions were found to be 30 °C, pH 7.0, fructose as the preferred carbon source, and yeast extract as the optimal nitrogen source. Nutritional analysis revealed that the fruiting bodies were rich in protein (15.4 g/100 g dry weight), dietary fiber (34.7 g/100 g dry weight), and minerals, while being low in fat (3.5 g/100 g dry weight). The most abundant amino acids were glutamic acid, followed by aspartic acid. The polysaccharides exhibited significant antioxidant activity, with ABTS^+^ scavenging comparable to that of Vitamin C (Vc), achieving a clearance rate of 96.95% at concentrations between 0.25–5.00 mg/mL. At a concentration of 5 mg/mL, the DPPH and OH radical scavenging activities reached their peak (83.77% and 67.31%, respectively), along with the highest iron ion reducing capacity (FRAP value: 4.43 mmol/L. Polysaccharides also exhibited notable anticancer activity, inhibiting HepG2 liver cancer cells and MDA-MB-468 breast cancer cells, with IC_50_ values of 3.896 mg/mL and 2.561 mg/mL, respectively. This study demonstrates that wild *H. coralloides* can be successfully cultivated in vitro. In conclusion, the fruiting bodies possess substantial nutritional value, and the polysaccharides extracted from them show promising antioxidant and anticancer activities, particularly against HepG2 liver cancer cells and MDA-MB-468 breast cancer cells.

## 1. Introduction

*Hericium coralloides* (Scop.) Pers., a member of the Basidiomycota phylum, Agarico-mycetes class, Russulales order, and Hericiaceae family, is closely related to *Hericium erinaceus* (Bull.) Pers. [1]. This species, resembling coral in appearance, features a bright white coloration with a narrow base and coral-like branching, with fine branches densely covered with spines. *H. coralloides* is primarily found on the trunks or decayed wood of broad-leaved trees in Northeast, Northwest, and Southwest China [2]. As a rare edible and medicinal fungus, it is valued for its substantial nutritional and pharmacological properties.

*H. coralloides*, a rare edible and medicinal fungus, has attracted significant attention due to its unique flavor and abundance of bioactive compounds. Studies have shown that *H. coralloides* is rich in various bioactive substances and possesses notable physiological functions, including antitumor [3], immunomodulatory [4], antioxidant [5], anticoagulant, and cholesterol-lowering effects [6]. Furthermore, it can alleviate Alzheimer’s disease and cognitive dysfunction by activating the Nrf2 signaling pathway and modulating the gut microbiota [7,8]. These remarkable physiological functions highlight its considerable potential for application in functional food development and the discovery of novel drug lead compounds, underscoring its global research and development value.

Due to its appealing appearance and rich nutritional and medicinal values, *H. coralloides* has gained considerable popularity among consumers in recent years, with promising prospects for industrial development. Like other edible fungi, such as black fungus, shiitake mushrooms, and reishi, *H. coralloides* is a wood decaying fungus that primarily utilizes lignocellulose as its nutritional source. Research into selecting superior strains and developing novel cultivation substrates for *H. coralloides* has made significant strides, resulting in technological breakthroughs. Large-scale production of this highly nutritious and delicious fungus is anticipated in the near future, offering it to a broader consumer base. However, current research on wild *H. coralloides* exhibits significant regional limitations both domestically and internationally. All reported strains so far have been isolated from Northeast China regions such as the Greater and Lesser Khingan Mountains and the Changbai Mountains [9]. In contrast, the resources from the unique growth environment of the Qinghai–Tibet Plateau remain entirely uninvestigated. The extreme conditions of the Tibetan Plateau (e.g., high UV radiation, low temperatures, and hypoxia) may have driven local strains to evolve distinct metabolic pathways, potentially leading to the synthesis of compounds with enhanced bioactivities. This endows these strains with significant development potential not found in strains from other regions [10].

To address this limitation and fill the resource gap in this critical region, this study successfully isolated and domesticated a wild strain of *H. coralloides* from Tibet, obtaining its fruiting bodies. On this basis, we systematically determined the fundamental nutritional composition of the fruiting bodies and focused on evaluating the in vitro antioxidant activity of its polysaccharides and their inhibitory effects on the proliferation of HepG2 and MDA-MB-468 tumor cells. This research aims to provide new germplasm material and a scientific basis for the resource conservation and utilization of this rare fungus, while also laying the foundation for exploring innovative drugs and functional foods derived from microorganisms inhabiting unique environments.

## 2. Materials and Methods

### 2.1. Experimental Materials

The experimental material was a wild fruiting body sample, numbered SH001, collected from Qinduo Town, Bomi County, Tibet Autonomous Region, from which mycelium was obtained after tissue separation.

### 2.2. Main Reagents

Maltose, mannose, fructose, soluble starch, sucrose, glucose, tryptone, yeast extract, beef extract, copper sulfate, potassium sulfate, anhydrous ether, anhydrous ethanol, acetic acid, sulfuric acid, sodium acetate, sodium hydroxide, potassium nitrate, ammonium nitrate, urea, magnesium sulfate, Vitamin B1 (V_B1_), potassium dihydrogen phosphate, hydrochloric acid, 2-mercaptoethanol, and other reagents (analytical grade, purchased from China National Pharmaceutical Group Corporation (Beijing, China)). D3390-01 Fungal DNA Kit (OMEGA Bio-Tek, Norcross, GA, USA); 2,2-Diphenyl-1-picrylhydrazyl (DPPH) 2,2′-Azino-bis (3-ethylbenzothiazoline-6-sulfonic acid (ABTS), 2,4,6-Tripyridyl-s-triazine (TPTZ) were obtained from a supplier located in Beijing, China. and ascorbic acid (St. Louis, MO, USA). Cell culture medium (Logan, UT, USA). Fetal bovine serum (Gibco, Grand Island, NY, USA).

### 2.3. Experimental Culture Medium

Enriched PDA Medium: 200 g of peeled potatoes, 20 g of glucose, 5 g of peptone, 2 g of K_2_HPO_4_, 1.5 g of MgSO_4_, 10 mg of V_B1_, 20 g of agar, and 1 L of distilled water, adjusted to natural pH.

Liquid Inoculum Culture Medium: 200 g of peeled potatoes, 20 g of fructose, 2 g of ammonium sulfate, 1.5 g of magnesium sulfate, 2 g of potassium dihydrogen phosphate, 10 mg of V_B1_, and 1 L of distilled water, pH 5.0.

Substrate for Cultivation: 60% hardwood sawdust, 20% cottonseed hulls, 18% wheat bran, 1% lime, and 1% sugar, mixed in the appropriate proportions with water to achieve a final moisture content of 60%.

### 2.4. Morphological Identification of Strain SH001

The morphological characteristics of both the fruiting body and the mycelium of strain SH001 were examined. The observations were made with reference to the work of Li et al. [1].

### 2.5. Molecular Identification of Strain SH001

The mycelium DNA of strain SH001 was extracted using the OMEGA Fungal DNA Extraction Kit. The extracted DNA was used as a template for PCR amplification with the universal fungal primers ITS1/ITS4, following the amplification and sequencing protocols described by Liu et al. [11]. After amplification, 3 μL of the PCR product was subjected to 1% agarose gel electrophoresis, and a single bright band was observed. The remaining PCR product was then sent to Fuzhou Baijing Biotechnology Co., Ltd. (Fuzhou, China) for sequencing. The resulting ITS sequence was submitted to the NCBI Nucleotide Database (http://www.ncbi.nlm.nih.gov accessed on 16 October 2024) for BLAST (https://blast.ncbi.nlm.nih.gov accessed on 16 October 2024) analysis. High homology ITS sequences were downloaded, and a phylogenetic tree was constructed using MEGA 11 software to determine the taxonomic classification of the strain.

### 2.6. Biological Characterization of Strain SH001

#### 2.6.1. Effect of Carbon Sources on Mycelial Growth

Different carbon sources, including glucose, sucrose, fructose, maltose, mannose, and starch, were used to replace glucose in the enriched PDA medium, while keeping all other components constant. Activated fungal cultures were inoculated by making a 5 mm hole at the edge of the agar plates, and small fungal blocks were transferred to the center of the plates containing media with different carbon sources (9 cm diameter). The plates were incubated in the dark at 25 °C. The colony diameter was measured using the “cross” method. Each treatment was performed in five replicates.

The mycelial growth rate (mm/day) was calculated as:

Mycelial Growth Rate (mm/day) = (Colony Diameter (mm) − Inoculum Diameter (mm))/Growth Duration (d).(1)

#### 2.6.2. Effect of Nitrogen Sources on Mycelial Growth

Different nitrogen sources, including urea, yeast extract, ammonium sulfate, beef extract, peptone, and ammonium nitrate, were used to replace peptone in the enriched PDA medium, with all other components remaining constant. Fungal blocks were inoculated at the center of 9 cm diameter plates containing media with different nitrogen sources. The procedure was carried out as described for the effect of carbon sources on mycelial growth.

#### 2.6.3. Effect of pH on Mycelial Growth

Enriched PDA medium was used to investigate the effect of pH on mycelial growth. The pH was adjusted to values of 5.0, 6.0, 7.0, 8.0, 9.0, and 10.0 using 1.0 mol/L NaOH and 1.0 mol/L HCl solutions. The procedure was conducted as described for the effect of carbon sources on mycelial growth.

#### 2.6.4. Effect of Temperature on Mycelial Growth

Enriched PDA medium was used to investigate the effect of temperature on mycelial growth. Following inoculation, the plates were incubated in constant temperature incubators at 15 °C, 20 °C, 25 °C, 30 °C, 35 °C, and 40 °C under dark conditions. The procedure was performed as described for the effect of carbon sources on mycelial growth.

### 2.7. Domestication and Cultivation Trials of H. coralloides

The liquid mycelial culture medium was aliquoted into Erlenmeyer flasks, with 100 mL per flask, and sterilized in a high-temperature, high-pressure autoclave for future use. Under a sterile laminar flow hood, PDA agar plugs (7 mm in diameter) were inoculated into shake flasks, with six plugs per flask, and sealed with stoppers. The flasks were then incubated in the dark at 23 °C with shaking at 160 rpm.

Cottonseed hulls were fully soaked in water for 12 h, then thoroughly drained. A mixture of fermented wood sawdust, pre-moistened cottonseed hulls, wheat bran, lime, and white sugar was prepared in appropriate proportions. Water was added incrementally and mixed thoroughly to ensure the substrate absorbed sufficient moisture, maintaining a moisture content of approximately 60%. The pH was adjusted to 5.0. The substrate was aliquoted into cultivation bags, with 900 g (wet weight) per bag, and sterilized in an autoclave at 121 °C for 3 h. After cooling to room temperature, the bags were transferred to a sterile laminar flow hood, where liquid mycelial culture was carefully inoculated, with each flask inoculating five bags. Following inoculation, the bags were placed in a mycelial growth chamber and incubated at 22–25 °C under dark conditions for mycelial colonization.

After the mycelium has fully colonized the substrate and undergone a one-week maturation period, the bags were promptly transferred to the fruiting room for bag opening and fruiting induction. The temperature was maintained at 18–20 °C, and the relative humidity was increased to 95%. Once the primordia appeared, the temperature was adjusted to 20–23 °C to promote further maturation and differentiation of the fruiting bodies.

### 2.8. Nutritional Composition Analysis

#### 2.8.1. Moisture Content Determination

The moisture content was determined using the direct drying method. An appropriate amount of fruiting body was weighed (W_1_) in a pre-weighed dish (W_0_), dried at 70 °C until constant weight was achieved, cooled in a desiccator, and reweighed (W_2_). The calculation formula is as follows:

Moisture content (g/100 g) = [(W_1_ − W_2_)/(W_1_ − W_0_)] × (100 g/100 g) (2)

#### 2.8.2. Protein Content Determination

The protein content was measured according to the Kjeldahl method as referenced by Amin et al. [12]. The fruiting body was weighed and digested with a potassium sulfate–copper sulfate catalyst and concentrated sulfuric acid, heated at 380–420 °C until a clear solution was obtained. After cooling, the mixture was alkalinized, distilled, and the released ammonia was absorbed in boric acid. The solution was titrated with 0.01 mol/L HCl standard solution to the endpoint, and the volume difference was recorded. Blank controls and instrument calibration were performed. The calculation formula is as follows:

Nitrogen content (g/100 g) = [(V_sample_ − V_blank_) × C × 14.01/_msample_] × 100 (3)

Protein content = Nitrogen content × 6.25(4)

#### 2.8.3. Ash Content Determination

Ash content was determined using the high-temperature incineration method as described by Uffelman et al. [13] An appropriate amount of sample (precisely 0.001 g) was weighed into a pre-weighed crucible, carbonized until smokeless, and incinerated in a muffle furnace at 550 °C for 4–6 h until gray-white ash was obtained. The crucible was cooled to 200 °C in the furnace, transferred to a desiccator, and weighed at room temperature. The incineration was repeated until constant weight was achieved (weight difference ≤ 0.5 mg). Blank correction and desiccator hygroscopicity control were performed. The calculation formula is as follows:

Ash content (g/100 g) = [(ash + crucible weight − crucible weight)/sample mass] × 100.(5)

#### 2.8.4. Fat Content Determination

Fat content was measured following the Soxhlet extraction method as outlined by Amin et al. [12] The sample was crushed, dried, homogenized, and packed into filter paper thimbles, which were then placed in a Soxhlet extractor. Excess diethyl ether was added, and the system was refluxed for 10 h to dissolve fats. After extraction, the solvent was evaporated, and the fat-containing flask was dried to constant weight. The fat mass was measured gravimetrically.

Fat content (g/100 g) = [fat mass/sample mass × 100].(6)

#### 2.8.5. Total Sugar Content Determination

The sample was ground and passed through an 80-mesh sieve, and 5 g was weighed into a 100 mL volumetric flask. After adding 50 mL of water to dissolve the sample, petroleum ether was used for defatting, and the residue was transferred back to the volumetric flask. Subsequently, 5 mL of zinc acetate solution (21.9 g/100 mL) and 5 mL of potassium ferrocyanide solution (10.6 g/100 mL) were added, followed by magnetic stirring for 30 min. The solution was then diluted to the mark with water. After filtration through dry filter paper, the initial filtrate was discarded, and the subsequent filtrate was passed through a 0.45 μm membrane for further analysis. A sugar-specific amino column (250 × 4.6 mm, 5 μm) was used for separation, with a mobile phase of acetonitrile-water (70:30, *v/v*) at a flow rate of 1.0 mL/min and a column temperature of 40 °C. An evaporative light scattering detector (ELSD) was employed with a drift tube temperature of 85 °C and a nitrogen pressure of 350 kPa. A standard curve was constructed using glucose, fructose, sucrose, maltose, and lactose standards (0–10 mg/mL), and the total sugar content was calculated according to the formula [14].

Total sugar content (g/100 g) = [(ρ − ρ_0_) × V × n/(m × 1000)] × 100 (7)

(ρ: concentration of the sample solution, mg/mL; V: volume, mL; n: dilution factor; m: sample mass, g)

Key Supplementary Note:

This analysis, conducted in accordance with Method 1 of GB 5009.8-2016 [15], specifically quantifies free monosaccharides (glucose, fructose) and disaccharides (sucrose, maltose, lactose). It explicitly excludes polysaccharide components, as the requisite acid hydrolysis steps were not performed.

#### 2.8.6. Dietary Fiber Content Determination

Dietary fiber content was quantified using the enzymatic gravimetric method as referenced by Phillips et al. [16]. The fruiting body was crushed and sequentially hydrolyzed with α-amylase, protease, and glucosidase (pH 8.2, 37 °C) to remove starch and proteins. The residue was precipitated with ethanol, filtered into a pre-weighed sintered glass crucible, washed with 78% ethanol and acetone, dried to constant weight at 105 °C, and weighed.

Dietary fiber content (g/100 g) = [(residue mass − blank)/sample mass] × 100.(8)

#### 2.8.7. Sodium Content Determination

Sodium content was determined using flame atomic emission spectrometry as per Moniruzzaman et al. [17]. The sample was precisely weighed (0.001 g), digested with nitric acid via microwave, diluted to volume, and filtered. Sodium standard solutions were prepared, and emission intensity was measured at 589 nm using flame atomic emission spectroscopy (FAES) to construct a calibration curve. The sample solution was analyzed identically.

Sodium content (g/100 g) = [(measured concentration × dilution factor × 0.1)/sample mass] × 100.(9)

#### 2.8.8. Amino Acid Content Determination

Amino acid content was analyzed using an automatic amino acid analyzer, as referenced by Purkiewicz et al. [18]. The sample was hydrolyzed with 6 mol/L hydrochloric acid at 110 °C for 24 h, diluted to volume, centrifuged, and filtered. The filtrate was injected into an amino acid analyzer, separated via ion-exchange chromatography, and subjected to post-column derivatization with ninhydrin. Peak areas of individual amino acids were detected. Calibration curves were generated using standard amino acid solutions.

Total amino acid content (g/100 g) = [∑(concentration of each amino acid × dilution factor × 0.1)/sample mass] × 100.(10)

Three parallel experiments were conducted for each analysis, and the average values of the respective indicators were calculated.

### 2.9. Exploration of the Bioactivity of H. coralloides Polysaccharides

#### 2.9.1. Preparation of Polysaccharides from *H. coralloides* Fruiting Bodies

The method described by Smiderle et al. [19] was slightly modified as follows: After freeze-drying, the fruiting bodies were ground into powder and sieved through an 80-mesh screen for use. A ratio of 1:20 (g:mL) of the sample to 75% ethanol was added, followed by ultrasonic treatment for 2 h. The filtrate was removed by suction filtration. Then, a ratio of 1:30 (g:mL) of the sample to distilled water was added and extracted in a 90 °C water bath for 2 h. The extraction was repeated twice, and the extracts were combined. The extract was concentrated in a rotary evaporator to one-third of its original volume. Anhydrous ethanol was added at a 4:1 (*v/v*) ratio, and the mixture was left at 4 °C overnight for alcohol precipitation. The solution was centrifuged at 5000 rpm for 10 min, and the precipitate was collected. The precipitate was re-dissolved in water and mixed with an equal volume of Sevage solution (chloroform–n-butanol = 4:1), followed by stirring and centrifugation at 10,000 rpm for 3 min. The supernatant was collected, and the process was repeated until no protein or other impurities were present. The polysaccharide solution was then dialyzed for 48 h against running water, and freeze-dried to a constant weight, yielding the crude polysaccharide freeze-dried powder from the fruiting bodies.

#### 2.9.2. Chemical Antioxidant Activity of *H. coralloides* Fruiting Body Polysaccharides

##### Hydroxyl Radical Scavenging Activity Assay

The method described by Ding et al. [20] was slightly modified as follows: Poly-saccharide solutions with concentrations of 0.025, 0.050, 0.250, 0.500, 1.000, 2.000, and 5 mg/mL, along with V_C_ solutions, were accurately prepared. In a 96-well plate, 75 μL of poly-saccharide solution at each concentration was added to the respective wells. Subsequently, 15 μL of FeSO_4_ solution (9 mmol/L), salicylic acid-ethanol solution (9 mmol/L), and H_2_O_2_ solution (8.8 mmol/L) were added sequentially to each well. The plate was gently shaken to ensure proper mixing. Finally, 100 μL of distilled water was added to each well. The plate was then incubated in a 37 °C water bath for 30 min, and absorbance at 510 nm was measured, recorded as Ay. V_C_ was used as a positive control. The reaction system for the blank group was prepared by replacing the polysaccharide sample with distilled water, and the absorbance was recorded as Ao. For the control group, distilled water was used instead of the H_2_O_2_ solution, and the absorbance was recorded as Ap. Five replicates were performed for each concentration, and the average values were calculated. The calculation formula is as follows:

Hydroxyl radical scavenging rate (%) = [1 − (Ay − Ap)/Ao] × 100(11)

##### ABTS^+^ Scavenging Activity Assay

The method described by Miller et al. [21] was slightly modified as follows: A 5 mL aliquot of 7.0 mmol/L ABTS solution and 88 μL of 2.45 mmol/L potassium persulfate aqueous solution were mixed and kept in the dark at room temperature for 12–16 h to prepare the ABTS+ stock solution. The stock solution was then diluted with distilled water, and its absorbance at 734 nm was measured using a spectrophotometer to adjust the absorbance to 0.70 ± 0.023, which was used as the ABTS+ working solution, prepared fresh for each use. In a 96-well plate, 100 μL of polysaccharide solutions at concentrations of 0.025, 0.05, 0.25, 0.5, 1, 2, and 5 mg/mL, along with the ABTS+ working solution, were added to each well and mixed thoroughly. The plate was incubated in the dark at 25 °C for 20 min, and absorbance at 734 nm was measured using a microplate reader (denoted as Ay). V_C_ was used as a positive control. For the blank group, the polysaccharide sample was replaced with distilled water, and the absorbance was recorded as Ao. In the control group, distilled water was used instead of the ABTS+ solution, and the absorbance was recorded as Ap. Five replicates were performed for each concentration. The calculation formula is as follows:

ABTS Radical Scavenging Rate (%) = [1 − (*A*_y_ − A_p_)/A_o_] × 100 (12)

##### DPPH Radical Scavenging Activity Assay

The method described by Saiga et al. [22] was slightly modified as follows: Aliquots of 100 μL of polysaccharide solutions (0.025, 0.050, 0.250, 0.500, 1.000, 2.000, and 5.000 mg/mL) and 0.2 mmol/L DPPH solution were added sequentially to a 96-well plate. The plate was gently shaken to ensure thorough mixing, and the reaction was allowed to proceed in the dark at room temperature for 30 min. Absorbance at 517 nm was then measured and recorded as Ay. V_C_ was used as a positive control. The blank group reaction system was prepared by replacing the polysaccharide sample with absolute ethanol, and the absorbance was recorded as Ao. In the control group, absolute ethanol was used instead of the DPPH solution, and the absorbance was recorded as Ap. Five replicates were performed for each concentration. The DPPH radical scavenging activity was calculated using the following formula:

DPPH Radical Scavenging Activity (%) = [1 − (A_y_ − A_p_)/A_o_] × 100 (13)

##### Ferric Ion Reducing Antioxidant Power (FRAP) Assay

The method described by Benzie et al. [23] was slightly modified as follows: A FRAP working solution was prepared by mixing 0.3 mol/L acetate-acetic acid buffer (pH 3.6), 0.02 mol/L FeCl_3_ solution, and 0.01 mol/L TPTZ solution in a volume ratio of 10:1:1, and used fresh. Aliquots of 0.5 mL of FeSO_4_ solutions at concentrations of 0.025, 0.1, 0.15, 0.2, 0.4, 0.5, 0.8, 1.0, and 1.5 mmol/L were added to 3.0 mL of the FRAP working solution. The mixtures were thoroughly mixed and incubated at 37 °C for 15 min. Absorbance at 593 nm was measured to construct a standard curve. The same procedure was used to determine the absorbance of each reaction system. Specifically, for the reaction mixtures containing polysaccharide solutions at concentrations of 0.025, 0.05, 0.25, 0.5, 1, 2, and 5 mg/mL, combined with the FRAP working solution, the absorbance at 593 nm was recorded as Ay. In the blank and control groups, distilled water replaced the polysaccharide sample and the FRAP working solution, with the corresponding absorbance values recorded as Ao and Ap, respectively. The FRAP value was calculated by determining the difference between Ay, Ao, and Ap, and then referring to the standard curve to obtain the corresponding FeSO_4_ concentration.

#### 2.9.3. Toxicity of *H. coralloides* Polysaccharides on Different Cancer Cells

The HepG2 and MDA-MB-468 cancer cell lines were cultured in cell culture medium at 37 °C with 5% CO_2_ in a humidified incubator for subculturing. Upon reaching the logarithmic growth phase and achieving 80–90% confluence, the cells were harvested for plating. The original culture medium was discarded, and the cells were washed three times with PBS (pH 7.4). Trypsin was then added to digest the cells for 30 s, followed by the addition of fresh culture medium to terminate the digestion. The cell suspension was centrifuged at 1000 rpm for 3 min, the supernatant was discarded, and the cell pellet was resuspended in culture medium to ensure even dispersion. The cell density was adjusted to 1 × 10^5^ cells/mL. A 200 μL aliquot of the cell suspension was added to each well of a 96-well plate, with 200 μL of PBS added to the outermost wells as blanks. The plate was incubated at 37 °C with 5% CO_2_ for cell attachment. Once the cells had fully adhered, the original medium was removed, and treatment with poly-saccharide was administered. The treatment groups included a polysaccharide group, a blank group, and a control group. The polysaccharide group received 200 μL of culture medium containing polysaccharides at different concentrations (0.025, 0.05, 0.25, 0.5, 1, 2, and 5 mg/mL). The control group received 200 μL of drug-free culture medium, and the blank group consisted of medium without cells, serving as the zero control. Each group was set up with five replicate wells. The plate was incubated at 37 °C with 5% CO_2_ for 24 h. After incubation, the culture medium was discarded, and 100 μL of culture medium containing 10% MTT was added to each well. The plate was incubated for an additional 4 h. Afterward, the medium was removed, and 150 μL of dimethyl sulfoxide (DMSO) was added to each well. The plate was gently shaken until the purple-brown precipitate was completely dissolved, and the absorbance at 490 nm was measured using a microplate reader. Cell viability at different polysaccharide concentrations was calculated using the following formula, and the IC_50_ value was determined.

Cell viability (%) = [(OD of polysaccharide group − OD of blank group)/(OD of control group − OD of blank group)] × 100.(14)

### 2.10. Statistical Analysis

Data were analyzed with SPSS 26.0 and GraphPad Prism 5.0 and are expressed as mean ± SD. Significance was assessed by one-way ANOVA followed by the LSD post hoc test. Statistical significance was defined as *p* < 0.05.

## 3. Results

### 3.1. Identification of Wild H. coralloides Strain

#### 3.1.1. Morphological Identification

A wild *H. coralloides* specimen was collected from a broadleaf forest in Mainling County, Nyingchi City, Tibet. The wild fruiting body was white, with a long and thick stalk, and coral-like branches. The cap surface was rough, exhibiting radial grooves that extended outward up to 8 cm and reached a width of 10 cm and a height of 5 cm. Upon comparison, these characteristics were consistent with the description of *H. coralloides* in the “Illustrated Guide to Major Fungal Resources in China” [1].

#### 3.1.2. Molecular Identification

**Figure 1:**
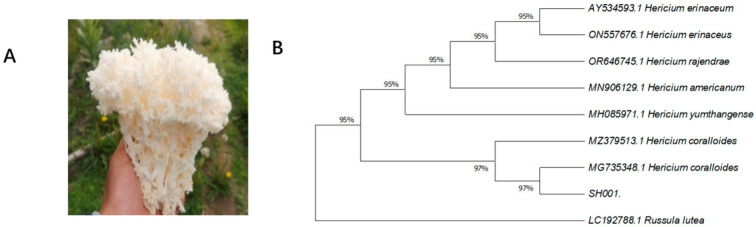
(**A**) Ecological photo of *H. coralloides*. (**B**) phylogeny based on ITS sequence construction.

The ITS sequence of strain SH001 (NCBI accession number: PQ094906) was compared for homology using GenBank. The results showed that the ITS sequence of strain SH001 shared 98.93% homology with the reported sequence of Hericium coralloides (MG735348). Using *Russula* lutea as the outgroup, ITS sequences of other *Hericium species* were included, and a phylogenetic tree was constructed using the neighbor-joining method. The tree (Figure 1) indicated that strain SH001 grouped closely with *H. coralloides* and showed a considerable genetic distance from other *Hericium species*. Based on the combined results from both morphological observation and molecular biology identification, the tested strain SH001 was confirmed to be *H. coralloides*.

### 3.2. Effects of Culture Conditions on H. coralloides Mycelial Growth

#### 3.2.1. Optimization of Culture Conditions

##### Carbon Source

**Figure 2:**
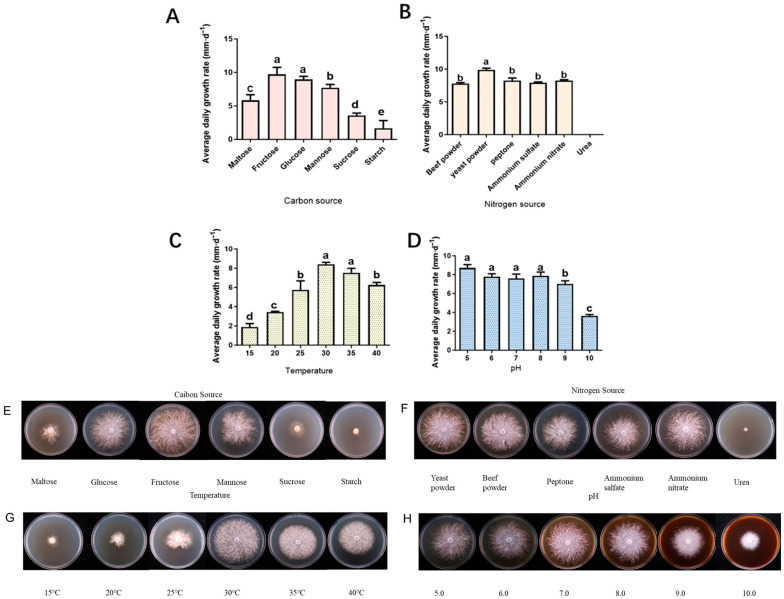
(**A**–**E**) Title of the Figure, with panel (**A**) providing a data visualization of the results shown in panel (**E**). (**B**–**F**) Title of the Figure, with panel (**B**) providing a data visualization of the results shown in panel (**F**). (**C**–**G**) Title of the Figure, with panel (**C**) providing a data visualization of the results shown in panel (**G**). (**D**–**H**) Title of the Figure, with panel (**D**) providing a data visualization of the results shown in panel (**H**). On the bar chart (**A**–**D**), the same letters indicate no significant difference, while different letters indicate a significant difference (*p* < 0.05).

The mycelium of *H. coralloides* SH001 can grow on various carbon sources, but significant differences in growth were observed. The mycelium exhibited the fastest growth when cultured on fructose, showing a white, dense, and robust appearance, with optimal growth performance. In contrast, starch resulted in the poorest growth, significantly lower than the control group. The growth rates, in descending order, were: fructose (10.15 mm/d) > glucose (8.81 mm/d) > mannose (7.61 mm/d) > maltose (5.63 mm/d) > sucrose (3.53 mm/d) > starch (1.31 mm/d) (Figure 2A). Overall, fructose is identified as the most suitable carbon source for the mycelial growth of *H. coralloides*.

##### Nitrogen Source

The mycelium of SH001 was capable of growth on various nitrogen sources, with noticeable differences in growth performance. The fastest growth occurred when cultured with yeast extract, and the growth rates were ranked from highest to lowest as follows: yeast extract (9.85 mm/d) > ammonium nitrate (8.25 mm/d) > peptone (8.23 mm/d) > ammonium sulfate (7.93 mm/d) > beef extract (7.78 mm/d) (Figure 2B). No growth was observed when urea was used as the nitrogen source. Overall, yeast extract was the most effective nitrogen source for promoting robust, dense, and healthy mycelial growth in *H. coralloides*.

##### pH

The mycelium of *H. coralloides* SH001 can grow within a pH range of 5.0 to 10.0. The fastest growth rate was observed at pH 5, with a growth rate of 8.71 mm/d (Figure 2D). No significant difference in growth rates was found between pH 6 and pH 7 (*p* > 0.05), though the mycelium at pH 7 appeared denser and more compact than at pH 5.0. The growth rate at pH 5.0 was significantly slower compared to pH 9 and pH 10 (*p* < 0.01). Based on the growth rate and overall performance, pH 7 was found to be the most suitable for optimal mycelial growth.

##### Temperature

*H. coralloides* SH001 does not tolerate low temperatures and cannot grow at 15 °C. The growth rate of the mycelium increased progressively with temperature. The growth rates, from highest to lowest, were: 30 °C > 35 °C > 40 °C > 25 °C > 20 °C > 15 °C. The mycelium grew fastest at 30 °C (8.40 mm/d) and 35 °C (7.10 mm/d), followed by 40 °C (6.25 mm/d) (Figure 2C). At 30 °C, the mycelium was the densest and exhibited well-defined edges, while at 35 °C, the mycelium was also dense, but with slightly less defined edges. In conclusion, the optimal temperature for mycelial growth of *H. coralloides* SH001 is 30 °C.

### 3.3. Domestication of H. coralloides

**Figure 3:**
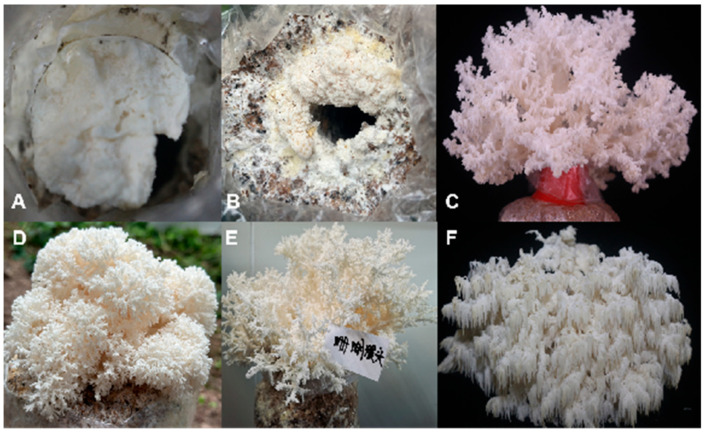
(**A**) *H. coralloides* are full of mycelium. (**B**–**F**) Artificial domestication of fruiting bodies.

After 28 d, the mycelium had fully colonized the bags. Following an additional 10 d under optimal environmental conditions, pale yellow primordia began to appear. The temperature was maintained between 20 and 23 °C, with humidity exceeding 90%. Ten days after the bags were opened, the fruiting bodies reached maturity and were harvested. The average fresh weight of the first flush of fruiting bodies per bag was 249.07 g, with a second flush also emerging. The growth conditions of the cultivated fruiting bodies are illustrated in Figure 3. As shown, both the fruiting bodies and mycelium of *H. coralloides* SH001 were white (Figure 3). The fruiting bodies displayed coral-like branches, which further branched into small twigs, from which dense, small spines developed.

### 3.4. Nutrient Content of H. coralloides Fruiting Bodies

Routine nutritional analysis (Table 1) showed that the contents of crude protein, ash, fat, total sugars, dietary fiber, and sodium in *H. coralloides* were 15.4 g/100 g dry weight, 6.8 g/100 g dry weight, 3.5 g/100 g dry weight, 1.6 g/100 g dry weight, 34.7 g/100 g dry weight, and 10.0 mg/100 g dry weight, respectively. Although the protein content of *H. coralloides* is lower than that of its congeneric species *H. erinaceus*, it still falls within the typical range (8–24%) observed in eight common edible mushrooms (shiitake, oyster, enoki, white button, velvet, king oyster, tea tree, and black fungus) [24]. The fat content is within the general range (2–4%) for edible mushrooms [25], and is lower than that found in *H. erinaceus*. The crude ash content in wild edible mushrooms, which consists of inorganic salts and heavy metals, serves as an indicator of the heavy metal content in the soil of the mushroom’s growing environment [26] and the contribution of minerals to nutritional value. The ash content of *H. coralloides* is comparable to that of *H. erinaceus*. Many provide nutritional advantages, such as a rich content of minerals. The crude fiber content of *H. coralloides* is slightly higher than that of the common mushroom Pleurotus eryngii [27], Fiber content varies substantially across edible mushroom species [28]. Sodium, an essential trace mineral for humans, cannot be synthesized in the body and must be obtained through food or water. It plays a vital role in muscle and nerve tissue. The sodium content of *H. coralloides* is 10.0 mg/100 g, suggesting that supplementation with trace minerals via fungi could be beneficial.

### 3.5. Amino Acids

**Figure 4:**
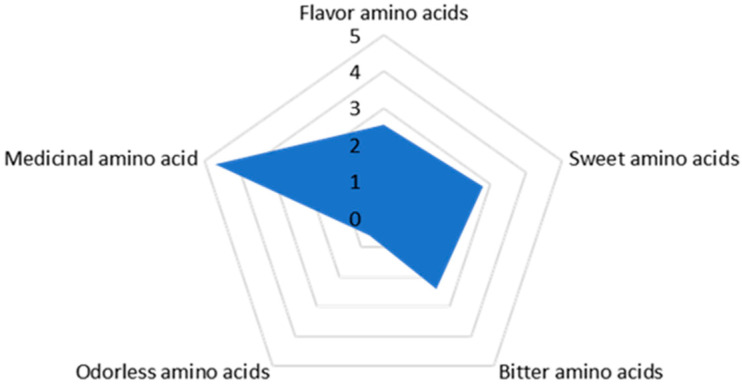
Random forest map of amino acids in *H. coralloides*.

The radar chart illustrates the amino acid composition of *H. coralloides* in (Figure 4). The concentration of medicinal amino acids was the highest (4.69 g/100 g dry weight), followed by sweet-tasting amino acids (2.81 g/100 g dry weight), umami amino acids (2.56 g/100 g dry weight), bitter amino acids (2.40 g/100 g dry weight), and neutral amino acids (0.61 g/100 g dry weight). The sweet-tasting amino acids primarily contribute to the flavor of *H. coralloides*, though the presence of sweet and salty compounds often masks the bitterness, ultimately defining the flavor profile. The most abundant amino acids are glutamic acid, followed by aspartic acid, which enhance the umami flavor of the mushroom. Arginine, a medicinal amino acid, is also essential for children’s healthy development. Overall, the amino acid profile and composition align with that of a high-quality protein source.

Fifteen amino acids were detected in the *H. coralloides* samples, with cysteine (Cys), methionine (Met), and tryptophan (Trp) not detected (Table 2). This suggests that either *H. coralloides* contains no measurable levels of Cys, Met, and Trp, or that their concentrations are too low to be detected by the instrumentation. The ratio of essential amino acids to total amino acids in *H. coralloides* was 0.32, while the ratio of essential to non-essential amino acids was 0.47. The E/T and E/N values are close to the ideal values for high-quality proteins recommended by the Food and Agriculture Organization/World Health Organization (approximately 0.40 and ≥ 0.60, respectively) [30].

### 3.6. Antioxidant Activity of H. coralloides Polysaccharide

**Figure 5:**
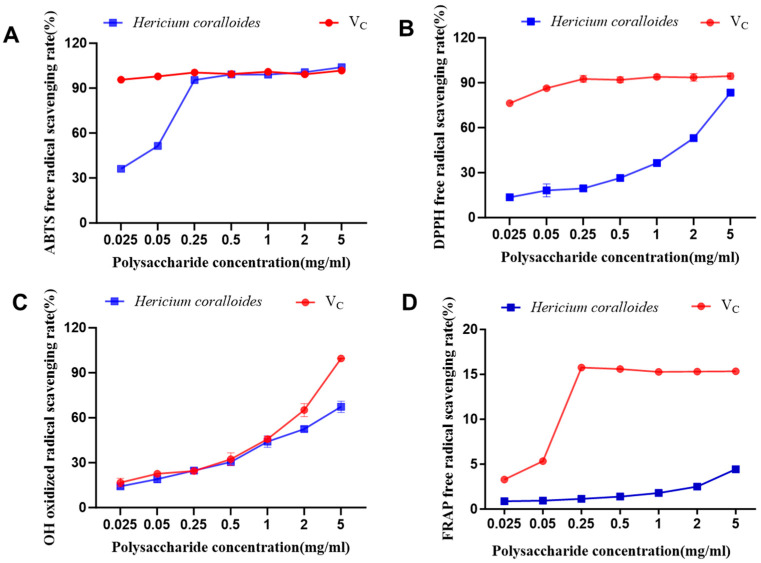
Antioxidant activity in vitro of polysaccharide from *H. coralloides*. (**A**) DPPH free radical clearance ability. (**B**) ABTS free radical clearance ability. (**C**) OH free radical clearance ability. (**D**) Ferric reducing antioxidant power. V_C_ is a positive control.

The antioxidant capacity of *H. coralloides* polysaccharides is illustrated in Figure 5. As shown in Figure 5A, at a concentration of 0.25 mg/mL, the ABTS radical scavenging rate reached 96.95%, which is comparable to the positive control group. Within the concentration range of 0.25–5.00 mg/mL, the scavenging activity was similar to that of ascorbic acid (V_C_), demonstrating significant antioxidant activity with an EC_50_ value of 0.04 mg/mL. Figure 5B indicates that as the concentration increased, the difference in DPPH radical scavenging activity between the sample and the positive control (V_C_) gradually decreased. The DPPH radical scavenging ability exhibited a dose-dependent enhancement, reaching a peak of 83.77% at 5 mg/mL, with an EC_50_ value of 1.417 mg/mL. In Figure 5C, at polysaccharide concentrations ranging from 0 to 1 mg/mL, the scavenging ability against hydroxyl radicals did not differ significantly from that of the V_C_ group. However, as the concentration increased, the scavenging rate of hydroxyl radicals increased progressively, although it remained significantly lower than that of V_C_. The highest scavenging rate, 67.31%, was achieved at a concentration of 5 mg/mL, with an EC_50_ value of 2.655 mg/mL. Figure 5D demonstrates that, with increasing polysaccharide concentration, the ferric ion reducing power positively correlated with concentration, reaching a maximum FRAP value of 4.43 mmol/L at 5 mg/mL. However, at the same concentration, the reducing power was significantly lower than that of the V_C_ group.

### 3.7. In Vitro Anticancer Activity of H. coralloides Polysaccharides

Fungi are valuable biological resources [31], and with advancements in science and technology, the medicinal potential of fungal polysaccharides has attracted increasing attention. Fungal polysaccharides are noncytotoxic compounds that do not exhibit harmful side effects on normal cells [32]. They have demonstrated the ability to inhibit cancer cell proliferation [33] and possess antioxidant properties.

**Figure 6:**
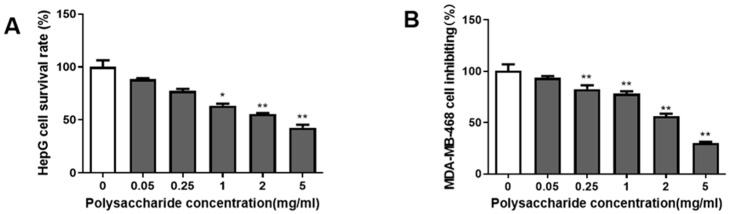
Effects of polysaccharides of *H. coralloides* on activities of different cancer cells. (**A**) HepG2 cell inhibition. (**B**) MDA-MB-468 cell inhibition. Compared to the control group (polysaccharide concentration is 0 mg/mL), * *p* < 0.05, ** *p* < 0.01.

To assess the anticancer effects of *H. coralloides* polysaccharides, an MTT assay was conducted to evaluate their toxicity on HepG2 and MDA-MB-468 cells (Figure 6). The results revealed that *H. coralloides* polysaccharides inhibited the growth of both cancer cell lines to some extent. A clear dose-dependent relationship was observed between polysaccharide concentration and cell viability. As the concentration of polysaccharides increased, the inhibitory effect on cancer cell growth became more pronounced. At a concentration of 5 mg/mL, the lowest cell viability was observed. The IC_50_ values for HepG2 and MDA-MB-468 cells were 3.896 mg/mL and 2.561 mg/mL, respectively, indicating that *H. coralloides* polysaccharides exerted a stronger inhibitory effect on MDA-MB-468 cells compared to HepG2 cells.

## 4. Discussion

Traditional fungal classification has primarily been based on the observation and description of morphological traits, which group fungi into different categories according to these features [34]. However, fruiting bodies may exhibit polymorphism influenced by environmental factors and nutritional conditions. In contrast, the ITS rDNA region evolves at a relatively rapid rate, displaying considerable sequence polymorphism. Furthermore, its short length facilitates amplification and sequencing, making it widely utilized in fungal taxonomy [35]. In this study, a combination of morphological observation and ITS sequence-based molecular identification was employed to characterize a wild strain (SH001) from Tibet. The results showed that the ITS sequence of this strain closely resembled that of strain MG735348.1, establishing a close phylogenetic relationship with *Hericium erinaceus*, and confirming its identification as *H. coralloides*. Additionally, the study observed that the strain exhibited accelerated mycelial growth at pH 5 and 30 °C when yeast extract was used as the nitrogen source and fructose as the carbon source.

Early studies have explored high-yield cultivation techniques for *Hericium coralloides*. Zhuang et al. found that a substrate consisting of 88% cottonseed hulls, 10% wheat bran, 1% gypsum, and 1% malic acid could increase the yield of *Hericium coralloides* to 150–200 g [36]. Zhang et al. conducted domestication and cultivation research on two wild *Hericium coralloides* strains from the Xiao Xing’anling region, demonstrating that a substrate containing 83% broadleaf sawdust, 15% wheat bran, 1% gypsum, and 1% sucrose achieved a fruiting body yield of 136.5 g, with a biological efficiency of 63.4% [37]. Through interviews with local farmers in Fujian, we selected suitable cultivation substrates, increasing the yield of *H. coralloides* to 249 g. This provides a new reference for cultivating *H. coralloides* in regions with similar climatic conditions to Fujian.

Research has demonstrated that proteins and peptides derived from mushrooms possess both nutritional and functional properties, conferring various health benefits such as antimicrobial, antiviral, antioxidant, anticancer, antihypertensive, angiotensin-converting enzyme (ACE) inhibitory, immunomodulatory, and enzymatic activities [38]. This study determined that the protein content in the fruiting bodies of *H. coralloides* was 15.4% (dry weight basis). Although slightly lower than that of its congeneric species *H*. *erinaceus*, this value remains within the typical protein content range (8–24%) reported for eight common edible mushroom species (including *Lentinula edodes*, *Pleurotus ostreatus*, *Flammulina velutipes*, *Agaricus bisporus*, *Xerocomus* sp., *Pleurotus eryngii*, *Agrocybe aegerita*, and *Auricularia auricula-judae*) and shows significant comparability with legume protein content [39]. Lipids in edible mushrooms are predominantly composed of unsaturated fatty acids, aligning with dietary recommendations for healthy fats. Hericium coralloides contains 3.5 g/100 g (dry weight) of total lipids, placing it at a moderate level among dried edible mushrooms (cf. Shiitake mushroom: 4.77 g/100 g; King oyster mushroom: 1.5 g/100 g) [40]. More importantly, the lipid profile of mushrooms is characterized by a high proportion of unsaturated fatty acids, which are beneficial to human health. From a nutritional perspective, mushrooms represent a valuable food ingredient that combines high carbohydrate content with substantial dietary fiber, serving as fundamental components for energy supply and intestinal health [41]. With carbohydrate and dietary fiber contents of 64.3 g/100 g and 34.7 g/100 g (dry weight), respectively, *H. coralloides* demonstrates potential as a good energy source. Dietary fiber is well-established for its role in preventing obesity, cardiovascular diseases, cancer, and type II diabetes. Previous research has confirmed that the high dietary fiber content in Agaricus bisporus effectively reduces blood lipid levels and improves liver health through multiple mechanisms, including physical adsorption (e.g., of cholesterol and bile salts), chemical inhibition (e.g., of lipid-digesting enzymes), and cellular regulation [42]. Given the documented hypolipidemic effects of *H. coralloides*, investigating whether these benefits are associated with its high dietary fiber content represents a key focus of our ongoing research. Furthermore, the fruiting bodies contain 15 amino acids, including all six essential amino acids, with an essential-to-total amino acid ratio of 0.32. The relatively low levels of total sugars and lipids suggest that this mushroom species can be utilized as an ideal raw material for developing low-sugar, low-fat food products, thereby providing a solid foundation for the development of value-added processed products from *H. coralloides*.

The evaluation of the bioactivity of *H. coralloides* extracellular polysaccharides (EPS) revealed significant antioxidant capacity. Specifically, the determined half-maximal effective concentration (EC_50_) values were 1.417 mg/mL for 2,2-diphenyl-1-picrylhydrazyl (DPPH) radical scavenging, 0.04 mg/mL for 2,2′-azino-bis(3-ethylbenzothiazoline-6-sulfonic acid) (ABTS^+^) radical scavenging, and 2.655 mg/mL for hydroxyl radical scavenging. This antioxidant efficacy demonstrates a substantially higher potency compared to previously reported data. For instance, Tabibzadeh et al. [3] reported EC_50_ values of 4.12 mg/mL for DPPH and 2.83 mg/mL for ABTS^+^ in wild Iranian specimens. Notably, the cultivated strain in this study exhibited markedly superior ABTS radical scavenging activity (EC_50_ = 0.04 mg/mL), being approximately 70 times more potent than the reported wild sample. Furthermore, within the concentration range of 0.25–5.00 mg/mL, the scavenging effects of the obtained polysaccharides on DPPH and ABTS radicals were comparable to those of ascorbic acid (V_C_), a widely recognized potent antioxidant. These findings are of significant implications, as they confirm that successful domestication and cultivation can yield strains with enhanced bioactivity, underscoring the substantial application potential of these polysaccharides as natural antioxidants. The antioxidant efficacy of the *H. coralloides* polysaccharides obtained in this study reaches a level comparable to that of synthetic antioxidants such as V_C_, thereby providing a solid scientific foundation for their future development in functional foods, pharmaceuticals, and cosmetics.

Experimental evidence suggests that polysaccharides from the Hericium genus exhibit significant anticancer potential [43]. For instance, polysaccharides from *H. erinaceus* have shown anticancer activity against human liver cancer (HepG2), breast cancer (MCF-7), and colon cancer (HCT116) cells [44]. These polysaccharides effectively inhibit the proliferation and colony formation of SGC-7901 cells by inducing apoptosis in the S phase and arresting the cell cycle [45]. In line with these findings, our study demonstrates that *H. coralloides* EPS is capable of inducing apoptosis in human liver cancer (HepG2) and triple-negative breast cancer (MDA-MB-468) cells.

In this research, we isolated and identified a wild *H. coralloides* strain from Tibet, China. By optimizing the cultivation substrate, we significantly enhanced the yield of *H. coralloides* fruiting bodies. The analysis of the fruiting bodies’ nutritional composition, along with the antioxidant and anticancer properties of its polysaccharides, underscores the potential of *H. coralloides* as a promising dietary supplement for cancer therapy. However, further studies are necessary to investigate its time-dependent effects on cancer cell elimination and to validate its efficacy in vivo.

This study holds important implications for the large-scale conservation of wild fungal germplasm and the expansion of the global edible fungal resource database. Additionally, it contributes to the economic exploration of wild fungal germplasm and offers valuable insights for the future development of cancer therapies, potentially making a significant contribution to human health.

In summary, this study successfully demonstrates that extracellular polysaccharides from the artificially domesticated *H. coralloides* exhibit remarkable antioxidant capacity, surpassing that of certain wild strains and matching the efficacy of V_C_. Building on these findings, future research will focus on two primary directions: first, to elucidate the anticancer mechanisms of these polysaccharides, with particular emphasis on their molecular roles in inducing tumor cell apoptosis and regulating key signaling pathways; second, to establish a systematic large-scale cultivation framework by optimizing culture medium formulations (C/N ratio, trace elements, etc.), controlling growth conditions (temperature, pH, aeration), and experimenting with diverse cultivation substrates to achieve efficient and stable yields of *H. coralloides*. This will provide both the raw materials and technical foundation for developing functional foods or anticancer adjuvant therapeutics.

## Figures and Tables

**Table 1:** Content of nutritional components in fruiting bodies of *H. coralloides*.

Nutrient Composition	Content (g·100^−1^ g^−1^ Dry Weight)	
	*Hericium coralloides*	*Hericium erinaceus* [29]
Moisture * (g/100 g) (fresh)	91.68	-
Moisture ** (g/100 g) (dry)	10.0	6.2
Carbohydrate (g/100 g)	64.3	61.1
Crude protein (g/100 g)	15.4 ± 1.2	20.08
Ash (g/100 g)	6.8 ± 0.8	6.8
Fat (g/100 g)	3.5 ± 0.5	5.1
Total sugar (g/100 g)	1.6 ± 0.2	-
Na (mg/100 g)	10.0 ± 1.5	-
Dietary fiber (g/100 g)	34.7 ± 0.7	-
Energy (kcal/100 g)	280.1	374

* Represents the fresh weight measurement. ** Represented as Total Carbohydrates.

**Table 2:** Content of amino acids in fruitbodies of *H. coralloides*.

Amino Acid Composition	Content (g/100 g)		
	*Hericium coralloids*		Egg
EAAs (essential amino acids)	Ile	0.27	0.649
	Val	0.49	0.636
	Lys	0.46	0.846
	Met	-	0.327
	Leu	0.59	1.047
	Phe	0.41	0.652
	Thr	0.49	0.588
	Trp	-	0.187
NEAA (nonessential Amino acid)	Arg	0.46	0.743
	His	0.21	0.266
	Try	0.21	0.495
	Ala	0.82	0.658
	Pro	0.49	0.343
	Ser	0.54	0.905
	Glu	1.67	1.593
	Gly	0.47	0.394
	Asp	0.89	1.212
	Cys	-	0.499
	EAA	2.71	4.932
	NEAA	5.76	7.108
	TAA	8.47	12.04
	E/T	0.32	0.41
	E/N	0.47	0.69

## Data Availability

The ITS sequence of strain SH001 is openly available in the NCBI accession number: PQ094906. The original contributions presented in the study are included in the article and supplementary materials; further inquiries can be directed to the corresponding author.

## Supplementary Materials

The following supporting information can be downloaded at: https://www.mdpi.com/article/10.3390/jof11110785/s1, Figure S1: About the abstract diagram of *H. coralloides*.

## References

[1] Li Y., Li T.H., Yang Z.L., Bau T., Dai Y.C. (2015). Atlas of Chinese Macrofungal Resources.

[2] Mao Z.L. (2000). Large Fungi of China.

[3] Tabibzadeh F., Alvandi H., Hatamian-Zarmi A., Kalitukha L., Aghajani H., Ebrahimi-Hosseinzadeh B. (2024). Antioxidant activity and cytotoxicity of exopolysaccharide from mushroom *Hericium coralloides* in submerged fermentation. Biomass Convers. Bioref..

[4] Williams L.M., Berthon B.S., Stoodley I.L., Williams E.J., Wood L.G. (2023). Medicinal mushroom extracts from *Hericium coralloides* and *Trametes versicolor* exert differential immunomodulatory effects on immune cells from older adults in vitro. Nutrients.

[5] Zhang J., Zhang J., Zhao L., Shui X., Wang L.A., Wu Y. (2019). Antioxidant and anti-aging activities of ethyl acetate extract of the coral tooth mushroom, *Hericium coralloides* (Agaricomycetes). Int. J. Med. Mushrooms.

[6] Cheng Y.F., Han A.L., Yun S.J., Chang M.C., Meng J.L., Feng C.P. (2018). Hypocholesterolemic effects and mechanisms of polysaccharides from *Hericium coralloides*. J. Nutr..

[7] Guan Y., Wang C.Y., Li L.Z., Dai X.J., Liu Y., Hsiang T., Liu S.Y., Wang D. (2024). Structural characterization of *Hericium coralloides* polysaccharide and its neuroprotective function in Alzheimer’s disease. Int. J. Biol. Macromol..

[8] Guan Y., Shi D.Y., Wang S.M., Sun Y.Y., Song W.Y., Liu S.Y., Wang C.Y. (2023). *Hericium coralloides* Ameliorates Alzheimer’s Disease Pathologies and Cognitive Disorders by Activating Nrf2 Signaling and Regulating Gut Microbiota. Nutrients.

[9] Song J.L., Xin Y., Zhou Z.F., Kang X.P., Zhang Y., Yuan W.D., Yu B. (2025). Biological Characteristics and Domestication of a Wild *Hericium coralloides*. Horticulturae.

[10] Zhang J., Zhang W., Zhang H., Zhang W., He C., Yu H., Xin G. (2024). Integrated metabolomics and transcriptomics reveal metabolic alterations of Ophiocordyceps sinensis from different geographical regions. Food Biosci..

[11] Liu C.Y., Hao X.Y., Lin P.L., Huang H.C., Liu K., Wu X.P., Fu J.S. (2020). Identification of a Wild Cordyceps cicadae Strain and Comparison of Its Intracellular and Extracellular Polysaccharides’ Anti-Liver Cancer Activity. J. Northwest AF Univ. Nat. Sci. Ed..

[12] Amin R., Rahman S.S., Hossain M., Choudhury N. (2018). Physicochemical and Microbiological Qualities’ Assessment of Popular Bangladeshi Mango Fruit Juice. Open Microbiol. J..

[13] Uffelman C.N., Doenges K.A., Armstrong M.L., Quinn K., Reisdorph R.M., Tang M., Krebs N.F., Reisdorph N.A., Campbell W.W. (2023). Metabolomics Profiling of White Button, Crimini, Portabella, Lion’s Mane, Maitake, Oyster, and Shiitake Mushrooms Using Untargeted Metabolomics and Targeted Amino Acid Analysis. Foods.

[14] Chen H.Q., Chen H.Q., Lu H.Q., Tang X., Zhang H., Chen Y.Q., Chen W. (2021). Carbohydrate analysis of *Mortierella alpina* by colorimetry and HPLC–ELSD to reveal accumulation differences of sugar and lipid. Biotechnol. Lett..

[15] (2016). National Food Safety Standard—Determination of Fructose, Glucose, Sucrose, Maltose and Lactose in Foods.

[16] Phillips K.M., McGinty R.C., Couture G., Pehrsson P.R., McKillop K., Fukagawa N.K. (2021). Dietary fiber, starch, and sugars in bananas at different stages of ripeness in the retail market. PLoS ONE.

[17] Moniruzzaman M., Chowdhury M.A., Rahman M.A., Sulaiman S.A., Gan S.H. (2014). Determination of mineral, trace element, and pesticide levels in honey samples originating from different regions of Malaysia compared to manuka honey. BioMed Res. Int..

[18] Purkiewicz A., Stasiewicz M., Nowakowski J.J., Pietrzak-Fiećko R. (2023). The Influence of the Lactation Period and the Type of Milk on the Content of Amino Acids and Minerals in Human Milk and Infant Formulas. Foods.

[19] Smiderle F.R., Ruthes A.C., van Arkel J., Chanput W., Iacomini M., Wichers H.J., Van Griensven L.J. (2011). Polysaccharides from *Agaricus bisporus* and *Agaricus brasiliensis* show similarities in their structures and their immunomodulatory effects on human monocytic THP-1 cells. BMC Complement. Med. Ther..

[20] Ding L., Zhang X., Zhang J. (2021). Antioxidant Activity In Vitro Guided Screening and Identification of Flavonoids Antioxidants in the Extract from *Tetrastigma hemsleyanum* Diels et Gilg. Int. J Anal. Chem..

[21] Miller N.J., Rice-Evans C., Davies M.J., Gopinathan V., Milner A. (1993). A novel method for measuring antioxidant capacity and its application to monitoring the antioxidant status in premature neonates. Clin. Sci..

[22] Saiga A., Tanabe S., Nishimura T. (2003). Antioxidant activity of peptides obtained from porcine myofibrillar proteins by protease treatment. J. Agric. Food Chem..

[23] Benzie I.F., Strain J.J. (1996). The ferric reducing ability of plasma (FRAP) as a measure of “antioxidant power”: The FRAP assay. Anal. Biochem..

[24] Li T., Lu S.J., Sun J.M., Xu Z.Q., Qi J., Liu P., Huang J.Z. (2021). Nutritional Composition Analysis and Evaluation of 26 Commonly Sold Edible Mushrooms. J. China Edible Fungi.

[25] Zheng H.Y., Yuan T.T., Xie Y.J., Chun C.Q. (2016). Nutritional Analysis on Edible Mushroom and Algae. J. China Edible Fungi.

[26] Wen C.Y., Xu M., Yang Y.L., Zhang J., Nie K., Zhang J. (2021). Research Progress on Nutritional Components and Value Evaluation of Wild Edible Fungi in China. J. China Edible Fungi.

[27] Han C., Lin K., He Y., Zhang Q., Che Z.M., He Y.X., Xiang W.L. (2015). Optimization of dietary fiber extraction from residue of Pleurotus eryngii and exploration of monosaccharide composition. Food Sci. Technol..

[28] Deng Y.Y., You J.K., Hua R., Wang J., Yang L.M., Sun D.F. (2022). Nutritional Analysis of Three Common Wild *Boltus* spp. and Three Staple Artificial Edible Fungi. J. China Edible Fungi.

[29] Cohen N., Cohen J., Asatiani M.D., Varshney V.K., Yu H.T., Yang Y.C., Li Y.H., Mau J.L., Wasser S.P. (2014). Chemical composition and nutritional and medicinal value of fruit bodies and submerged cultured mycelia of culinary-medicinal higher Basidiomycetes mushrooms. Int. J. Med. Mushrooms.

[30] Pellett P.L., Young V.R. (1990). Commentary: Joint FAO/WHO expert consultation on protein quality evaluation Bethesda, MD, USA, 4–8 December 1989. Ecol. Food Nutr..

[31] Dávila G.L.R., Murillo A.W., Zambrano F.C.J., Suárez M.H., Méndez A.J.J. (2020). Evaluation of nutritional values of wild mushrooms and spent substrate of *Lentinus crinitus* (L.) Fr. Heliyon.

[32] Chang X.N., Chen X.F., Gong P., Liu M., Wang M.R., Wang X.J. (2022). Structural characterization, in vitro anti-oxidative effect and hypoglycemic activity of lentinan from Rongshui county. China Food Addit..

[33] Li Y.L., Jiang M., Tan M.Q. (2020). Research Progress on Pharmacological Value of Polysaccharides from Edible Fungi. J. Ningxia Agric. For. Sci. Technol..

[34] Justo A., Miettinen O., Floudas D., Ortiz-Santana B., Sjökvist E., Lindner D., Nakasone K., Niemelä T., Larsson K.-H., Ryvarden L. (2017). A revised family-level classification of the *Polyporales* (*Basidiomycota*). Fungal Biol..

[35] Zhou J.L., Zhu L., Chen H., Cui B.K. (2016). Taxonomy and Phylogeny of *Polyporus* Group *Melanopus* (Polyporales, Basidiomycota) from China. PLoS ONE.

[36] Zhuang L., Sun Z.H., Yu S.T., Guo X.X., Yang N. (2024). New cultivar ‘ZLsh-1’ of Hericium coralloides. Mycosystema.

[37] Zhang P., Shi L., Yu H.Y., Sheng C.G., Wang F., Wang Y.F. (2024). Domestication Cultivation Research for Two Wild *Hericium coralloides* Strains from Xiaoxing’an Mountains. North. Hortic..

[38] Ionescu M., Dincă M.-N., Ferdeș M., Zăbavă B.-Ș., Paraschiv G., Moiceanu G. (2025). Proteins from Edible Mushrooms: Nutritional Role and Contribution to Well-Being. Foods.

[39] Sar T., Larsson K., Fristedt R., Undeland I., Taherzadeh M.J. (2022). Demo-scale production of protein-rich fungal biomass from potato protein liquor for use as innovative food and feed products. Food Biosci..

[40] Gochhi M., Dash P., Chinara N., Sahoo H.P., Rai V.K., Halder J., Das C., Ghosh G., Rath G., Kar B. (2025). A Narrative Review of the Nutritional Value and Biological Properties of Mushrooms. Curr. Drug Discov. Technol..

[41] Effiong M.E., Umeokwochi C.P., Afolabi I.S., Chinedu S.N. (2023). Assessing the nutritional quality of *Pleurotus ostreatus* (oyster mushroom). Front. Nutr..

[42] Zhao X., Zhang Y., Wang X., Yao L., Qu Y., Zhao F., Yun J. (2025). Structure, physicochemical properties, and hypolipidemic activity of soluble dietary fiber obtained from button mushroom (*Agaricus bisporus*). Food Chem. X.

[43] Hyder M.S., Dutta S.D. (2021). Mushroom-derived polysaccharides as antitumor and anticancer agent: A concise review. Biocatal. Agric. Biotechnol..

[44] Hetland G., Tangen J.-M., Mahmood F., Mirlashari M.R., Nissen-Meyer L.S.H., Nentwich I., Therkelsen S.P., Tjønnfjord G.E., Johnson E. (2020). Antitumor, Anti-inflammatory and Antiallergic Effects of *Agaricus blazei* Mushroom Extract and the Related Medicinal Basidiomycetes Mushrooms, *Hericium erinaceus* and *Grifola frondosa*: A Review of Preclinical and Clinical Studies. Nutrients.

[45] Zan X.Y., Cui F.J., Li Y.H., Yang Y., Wu D., Sun W.J., Ping L.F. (2015). *Hericium erinaceus* polysaccharide-protein HEG-5 inhibits SGC-7901 cell growth via cell cycle arrest and apoptosis. Int. J. Biol. Macromol..

