# Supplementary figures and images for: Domestication Cultivation and Nutritional Analysis of Hericium coralloides
Source: J Fungi (Basel). 2025 Oct 31;11(11):785. doi: 10.3390/jof11110785 (PMC12653868; doi:10.3390/jof11110785)

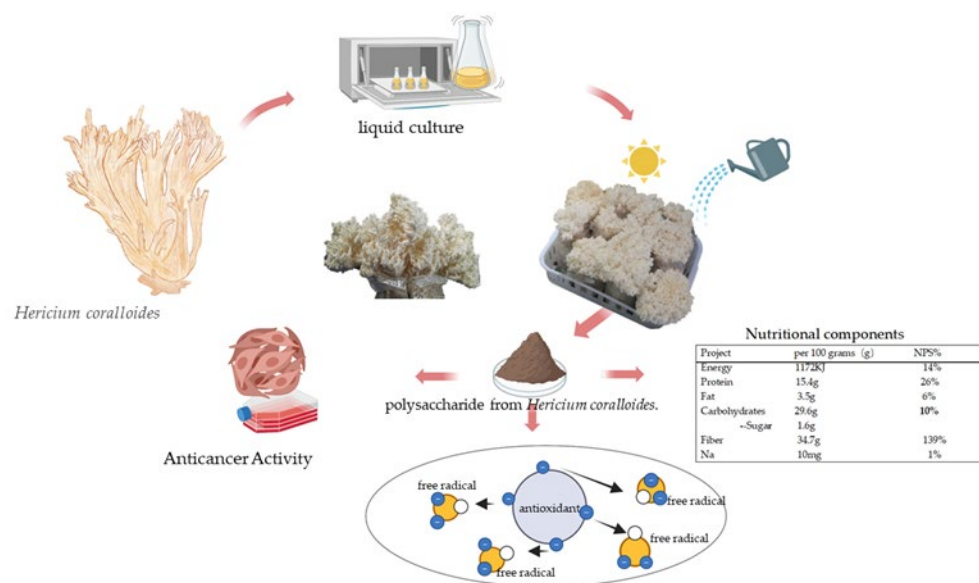

**Figure S1.** About the abstract diagram of *H. coralloides*

Supplement: Supplementary file 1 [file jof-11-00785-s001.zip › jof-3874263-supplementary.pdf]
